# Deleterious Effects of Caffeine Consumption on Reproductive Functions of Female Wistar Rats

**DOI:** 10.5935/1518-0557.20240055

**Published:** 2024

**Authors:** Eunice Ogunwole, Victor Oghenekparobo Emojevwe, Hannah Bolutife Shittu, Iyanuoluwa Elizabeth Olagoke, Favour Omolewami Ayodele

**Affiliations:** 1 Reproductive Physiology and Developmental Programming unit, Department of Physiology, University of Medical Sciences, Ondo City, Nigeria

**Keywords:** caffeine, infertility, oxidative stress, reproductive hormone, rats

## Abstract

**Objective:**

The deleterious effects of caffeine consumption on reproductive functions of female Wistar rats were investigated in this study.

**Methods:**

In this experimental study, 35 female Wistar rats (180-200g) were divided into 7 groups: Control, II-IV received oral caffeine (10, 20, and 40mg/kg/day respectively) for 21 days. V-VII received similar caffeine doses for 21 days, followed by a 21-day withdrawal period. The ovaries, fallopian tubes, and uteri were assessed for levels of malondialdehyde (MDA), nitric oxide (NO), reduced glutathione (GSH), superoxide dismutase (SOD), and catalase activity using spectrophotometry. Serum luteinizing hormone (LH), follicle-stimulating hormone (FSH), and estradiol levels were measured by ELISA. Organ histology was performed using microscopy. Statistical analysis employed ANOVA with significance at *p*<0.05.

**Results:**

Caffeine caused dose-dependent increases in MDA, NO, and catalase activity in the ovaries, fallopian tubes, and uteri which decreased upon withdrawal. GSH levels in the ovary and fallopian tubes decreased with caffeine intake but recovered during withdrawal. Caffeine reduced estradiol levels in a dose-dependent manner, its withdrawal led to reductions in serum LH at 20 and 40mg/kg/day and FSH at 40mg/kg/day. Histology revealed dose-dependent alterations in ovarian architecture with congested connective tissues. Caffeine caused sloughing of plicae in the muscularis of the fallopian tubes, degenerated epithelial layer in the uterus, and severe inflammation of the myometrial stroma cells that persisted during caffeine withdrawal.

**Conclusions:**

Caffeine consumption adversely impacted the female reproductive functions of rats, altering hormonal balance and organ structure which persisted even after caffeine withdrawal.

## INTRODUCTION

Human exposure to disruptive chemicals is linked to various reproductive dysfunctions, including infertility issues, miscarriages, and birth defects (Oluwole *etal.,* 2016; Vessa *et al.,* 2022; Dutta *et al.,* 2023; Lahimer *et al.,* 2023). Notably, approximately 16% of the general reproductive-age population faces fertility challenges (Sarkar & Gupta, 2016; Deshpande & Gupta, 2019). Among infertile couples, contributing factors are split roughly as follows: 46.6% female, 20% male, and the remaining 33.4% either caused by both genders or with no apparent cause (Deshpande & Gupta, 2019).

Caffeinated beverages have been implicated in fertility problems (Ogunwole *et al.,* 2015; Oluwole *et al.,* 2016; Lakin *et al.,* 2023). Caffeine is a unique nutritive constituent found in diverse products, including foods, dietary supplements, and drugs (Wierzejska, 2012; Doepker *et al.,* 2016; Reyes & Cornelis, 2018). It primarily comes from coffee (75%), tea (15%), and caffeinated sodas (10%), other sources include cocoa/chocolate products and various medications (Doepker *et al.,* 2016; Wierzejska *et al.,* 2019). With near-complete oral bioavailability and rapid absorption, caffeine exerts diverse biological effects, including central nervous system stimulation, increased catecholamine secretion, smooth muscle relaxation, and heart rate stimulation (Persad, 2011). Despite its widespread consumption and generally safe history, caffeine presents regulatory challenges due to its natural occurrence and use as an additive (Doepker *et al.,* 2016).

While moderate intake may have some cardiovascular and metabolic benefits, chronic exposure has been linked to various dysfunctions in human and animal models (Silletta *et al.,* 2007; Yuan *et al.,* 2021; Mendoza *et al.,* 2023). For example, excessive consumption of caffeinated energy drinks caused alteration in the auditory and visual relay center as well as other parts of the brain in animal models (Adjene *et al.,* 2014a; 2014b), it stimulated the secretion and production of gastrin and hydrochloric acid thereby affecting gastrointestinal tract (Nehlig, 2022) and interacting with the brain-gut axis negatively (Iriondo-DeHond *et al.,* 2020). Caffeine affects liver functions by altering the levels of liver enzymes (Heath *et al.,* 2017), accelerating time-related decline in renal function and augmented urinary protein excretion (Tofovic & Jackson, 1999) as well as reduction of renal function (Komorita *et al.,* 2022). Also, caffeine has been linked to an increased risk of lung cancer development (Ludwig *et al.,* 2014).

Notably, some epidemiological studies suggested associations between high prenatal caffeine consumption (around 300mg/day) and negative reproductive outcomes, including reduced fertility, fetal growth issues, and miscarriages (Cano-Marquina *et al.,* 2013; Lakin *et al.,* 2023). Given the limited research on how caffeine consumption and its withdrawal affects female reproductive function in rats, this study aims to investigate its potential impacts on female Wistar rats.

## MATERIALS AND METHODS

### Caffeine Preparation

Caffeine (Caffeine® Central Drug House Ltd. Corp. India) was freshly prepared by dissolving in distilled water and administered at 10, 20, and 40mg/Kg body weight with an oral cannula daily. The dosage regime was by the human study of Jensen *et al.* (2010) and experimental rats study of Oluwole *et al.* (2016).

### Experimental Animals

All procedures involving the use of animals conformed with the Animal Research: Reporting of in Vivo Experiments (ARRIVE) guidelines (NC3Rs Reporting Guidelines Working Group, 2010) and ethical standards of the University of Medical Sciences Animal Care and Use. This study employed thirty-five female Wistar rats, aged 12-14 weeks old (170-200 g body weight), obtained from the animal house of the University of Medical Sciences, Ondo city, Ondo state. All animals were housed in well-ventilated wire mesh cages under controlled laboratory conditions (temperature: 23±2°C; humidity: 55±5%; light/dark cycle: 12:12 hours) and acclimatized for two weeks. During this period, they had *ad libitum* access to standard laboratory rat chow and clean tap water.

### Experimental Design

Thirty-five adult female Wistar rats were grouped into seven (7), n = 5. Group I served as the control and received distilled water. Groups II-IV received daily oral doses of caffeine (10, 20, and 40mg/kg body weight, respectively) for 21 days. Groups V-VII received similar caffeine doses for 21 days, followed by a 21-day withdrawal period. The body weight of each rat was recorded once a week using an electronic digital weighing scale (EK5055, China). Additionally, body weight was measured on the day of sacrifice.

### Animal sacrifice and sample collection

Following the experimental procedures, the rats were euthanized by cervical dislocation. A midline incision was made along the linea alba, extending from the anterior abdominal wall to the thoracic cavity to expose the heart and internal organs. Blood was collected via cardiac puncture into plain serum bottles. After allowing the blood to clot for at least 45 minutes, samples were centrifuged at 3500 rpm for 15 minutes. The resulting supernatant (serum) was then carefully aspirated and stored at −20°C for subsequent hormonal assays. The ovaries, fallopian tubes, and uteri were then meticulously dissected, removing any adherent tissues. The weight of each organ was immediately measured using a digital electronic scale (model EHA501, China). Finally, the organs were homogenized for further biochemical analyses.

### Biochemical Analysis

Lipid peroxidation in the ovary, fallopian tube, and uterus was assessed by measuring malondialdehyde (MDA) levels using the method of Buege & Aust (1978). Nitric oxide (NO) levels were determined using the Griess reaction (Griess, 1879). Reduced glutathione (GSH) was quantified with a commercial spectrophotometric assay kit (Oxford Biomedical Research, USA). Tissue catalase and superoxide dismutase (SOD) activities were measured following the protocols described by Sinha (1972) and Misra & Fridovich (1976), respectively. Serum concentrations of follicle-stimulating hormone (FSH), luteinizing hormone (LH), and estradiol were determined using enzyme-linked immunosorbent assay (ELISA) kits (Fortress Diagnostics, UK) as described previously by (Emojevwe *et al.,* 2023).

### Histology

The ovaries fallopian tubes and uteri were fixed in Bouin’s fluid and processed for microscopic examination. The tissues were embedded in paraffin and sectioned to obtain a 4-5 µm-thickness with a microtome. The dewaxed sections were stained with hematoxylin and eosin and the slides were viewed under a light microscope at 400× magnification as previously described (Adjene *et al.,* 2014a).

### Statistical Analysis

Data were analyzed using GraphPad Prism Statistics software (version 8.0, USA). Results were presented as mean ± standard error of mean (SEM). The mean differences were compared by analysis of variance (one-way ANOVA). Statistical significance was set at *p*<0.05.

## RESULTS

### Effect of caffeine on the percentage change in body weight of female Wistar rats

**Figure 1:**
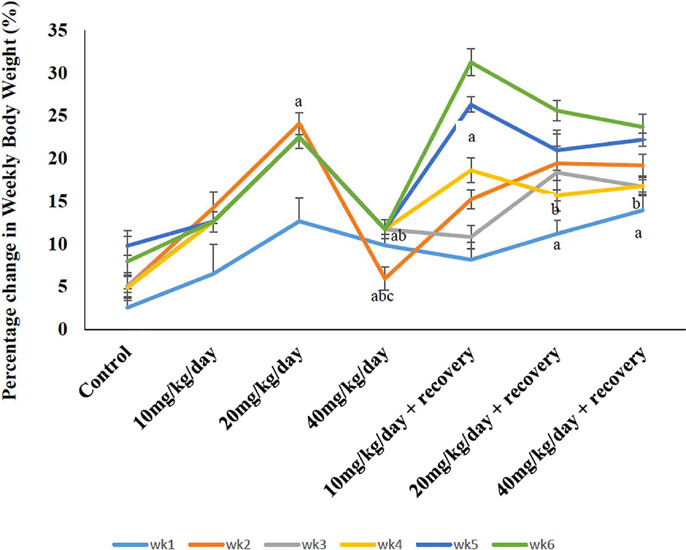
Effect of caffeine on the percentage change in body weight of female Wistar rats. Lines represents mean±SEM, n = 5, *p*<0.05. Week 1- ^a^*p*<0.05 compared to control. Week 2- ^a^*p*<0.05 compared to control, ^bc^*p*<0.05 compared to 10 and 20 mg/Kg/day, respectively. Week 3- ^a^*p*<0.05 relative to control, ^bc^*p*<0.05 relative to 10, and 20 mg/Kg/day. Week 4- ^a^*p*<0.05 relative to control, ^b^*p*<0.05 relative to 10mg/Kg/day.

The effect of caffeine on the percentage change in body weight of female Wistar rats is shown in Figure 1. The study revealed interesting patterns in body weight changes across the groups. During the first week, rats treated with both 20mg/kg/day and 40mg/kg/day caffeine exhibited significant weight gain (*p*<0.05) compared to the control group. However, in the second week, this trend shifted. While the 20mg/kg/day group continued to show a significant weight increase (*p*<0.05) compared to the control, the 40mg/kg/day group experienced a significant decrease (*p*<0.05) in body weight when compared to the control, 10mg/kg/day, and 20 mg/kg/day groups. Interestingly, the body weight of the 40mg/kg/day group reversed this trend in the third week, showing a significant increase (*p*<0.05) again. During the withdrawal phase (starting from the fourth week), the 10mg/kg/day caffeine-withdrawn group displayed a significant increase (*p*<0.05) in body weight compared to the control group. Conversely, both the 20mg/kg/day and 40 mg/kg/day caffeine-withdrawn groups experienced significant decreases (*p*<0.05) in body weight compared to the control group.

### Effect of caffeine on the relative organ weight of female Wistar rats

Table 1 shows the effects of caffeine on the relative organ weight of female Wistar rats. As shown, no significant changes were observed in the ovaries, fallopian tubes, and uterus of groups treated with 10 mg/kg/day, 20mg/kg/day, and 40mg/kg/day when compared with the control group during caffeine administration.

**Table 1:** Effect of caffeine on the relative organ weight of female Wistar rats.

Group	Control	10 mg/Kg/day	20 mg/Kg/day	40 mg/Kg/day	10 mg/Kg/day + Recovery	20 mg/Kg/day + Recovery	40 mg/Kg/day + Recovery
**Ovary**	0.10±0.02	0.06±0.01	0.07±0.01	0.07±0.00	0.07±0.01	0.08±0.01	0.10±0.01
**Fallopian tube**	0.24±0.05	0.2±0.048	0.17±0.02	0.14±0.01	0.15±0.02	0.18±0.03	0.20±0.02
**Uterus**	0.07±0.00	0.09±0.02	0.08±0.01	0.09±0.02	0.14±0.02	0.14±0.02	0.13±0.03

Data presented as mean ± SEM, n = 5.

### Effects of caffeine on the oxidant and antioxidant status of the ovary of female Wistar rats

The effects of caffeine on the oxidant and antioxidant status of the ovary of female Wistar rats are shown in Table 2. Accordingly, a significant decrease (*p*<0.05) in ovarian protein levels was observed in the 10 and 20mg/kg/day caffeine-treated groups compared to the control. However, the 40 mg/kg/day caffeine-withdrawn (recovery) group displayed a significant increase (*p*<0.05) in protein level. Caffeine treatment caused a significant increase (*p*<0.05) in ovarian MDA levels across all treated groups compared to the control. Conversely, during the withdrawal phase, MDA levels were significantly reduced (*p*<0.05) in all groups compared to the control and the 20 and 40mg/kg/day treated groups. All caffeine-treated groups displayed a significant increase (*p*<0.05) in ovarian NO levels during treatment. However, following withdrawal, NO levels became significantly reduced (*p*<0.05) in the 10 and 20mg/kg/day groups compared to the 10 and 40mg/kg/day caffeine-treated groups. No significant differences were observed in ovarian SOD levels among all groups compared to the control. Caffeine treatment significantly increased (*p*<0.05) catalase activity in the 40mg/kg/day group compared to the control. In contrast, withdrawal of caffeine caused a significant reduction (*p*<0.05) in catalase activities across all caffeine-withdrawn groups. Compared to the control, all caffeine-treated groups exhibited a significant decrease (*p*<0.05) in ovarian GSH levels. However, withdrawal of caffeine led to a significant increase (*p*<0.05) in GSH levels within the caffeine-withdrawn groups.

**Table 2:** Effect of caffeine on oxidant and antioxidant status of the ovary of female Wistar rats.

Group	Control	10mg/Kg/day	20mg/Kg/day	40mg/Kg/day	10 mg/Kg/day + Recovery	20 mg/Kg/day + Recovery	40 mg/Kg/day + Recovery
Protein level (g/dl)	4.27±0.35	2.90±0.06*	2.91±0.09*	3.36±0.27	3.87±0.25	3.92±0.13	4.13±0.40^ab^
MDA (U/mg)	0.63±0.18	4.95±0.43*	5.09±0.39*	5.66±0.32*	0.60±0.11^abc^	0.87±0.25^abc^	1.63±0.36^abc^
NO (U/mg)	5.24±0.24	9.64±0.25*	9.61±0.19*	9.80±0.19*	5.72±0.48^abc^	7.66±1.39	5.88±0.23^abc^
SOD (U/mg)	0.81±0.09	1.03±0.08	1.03±0.11	0.76±0.10	1.04±0.05	0.98±0.08	0.88±0.04
Catalase (IU/L)	11.6±0.30	20.25±4.20	25.6±6.44	30.12±4.74*	12.83±0.41^abc^	13.23±0.41^abc^	12.74±0.55^abc^
GSH (uM/mg)	1.47±0.11	0.70±0.01*	0.87±0.04*	0.86±0.05*	1.49±0.11^abc^	1.54±0.17^abc^	1.48±0.07^abc^

Data are presented as mean±standard error of the mean, n = 5, *p<0.05 compared with control, ^a^*p*<0.05 compared with 10mg/Kg/day caffeine, ^b^*p*<0.05 compared with 20 mg/Kg/day caffeine, ^c^*p*<0.05 compared with 40 mg/Kg/day caffeine.

### Effect of caffeine on oxidant and antioxidant status of the fallopian tube of female Wistar rats

As shown in Table 3, caffeine treatment had complex effects on the fallopian tubes. Protein levels only increased significantly (*p*<0.05) in the 40 mg/kg/day withdrawal group compared to the control. Malondialdehyde (MDA), a marker of oxidative stress, increased significantly (*p*<0.05) with 20 and 40 mg/kg/day treatment but dropped significantly (*p*<0.05) during withdrawal in all groups. Nitric oxide (NO) levels followed a similar pattern, with a significant decrease (*p*<0.05) only observed in the 40 mg/kg/day withdrawal group compared to treated rats. Superoxide dismutase (SOD), an antioxidant enzyme, displayed a rise (*p*<0.05) with 10 mg/kg/day caffeine but a decrease (*p*<0.05) in the 40 mg/kg/day withdrawal group compared to controls. Catalase activity mirrored this trend, increasing significantly (*p*<0.05) with higher caffeine doses (20 and 40 mg/kg/day) but decreasing significantly (*p*<0.05) after withdrawal. Finally, reduced glutathione (GSH), another antioxidant, exhibited a decrease (*p*<0.05) with all caffeine treatments, followed by a significant increase (*p*<0.05) in all withdrawal groups.

**Table 3:** Effect of caffeine on oxidant and antioxidant status of the fallopian tube of female Wistar rats.

Group	Control	10 mg/Kg/day	20 mg/Kg/day	40 mg/Kg/day	10 mg/Kg/day + Recovery	20 mg/Kg/day + Recovery	40 mg/Kg/day + Recovery
**Protein level (g/dl)**	3.75±0.30	3.24±0.15	3.29±0.28	3.05±0.13	3.73±0.15	4.01±0.48	4.2±0.43^c^
**MDA (U/mg)**	2.16±0.42	6.41±1.19	7.65±1.52*	7.62±1.01*	1.10±0.15^abc^	2.32±0.32^bc^	3.01±1.56^b^
**NO (U/mg)**	7.45±1.28	6.15±1.25	5.99±0.58	8.11±0.80	5.20±0.21	4.99±0.24	4.30±0.07^c^
**SOD (U/mg)**	0.35±0.07	1.14±0.06*	0.78±0.20	0.80±0.11	0.78±0.13	0.65±0.10	0.51±0.12^a^
**Catalase (IU/L)**	10.3±0.43	19.48±0.69	28.8±3.92*	20.0±3.54*	10.07±0.40^bc^	8.88±1.45^abc^	10.48±0.33^bc^
**GSH (uM/mg)**	1.91±0.12	0.68±0.02*	0.73±0.01*	0.77±0.03*	1.40±0.09*^abc^	1.61±0.15^abc^	1.44±0.10*^abc^

Data are presented as mean±standard error of mean, n = 5, *p<0.05 compared with control, ^a^*p*<0.05 compared with 10mg/Kg/day caffeine, ^b^*p*<0.05 compared with 20mg/Kg/day caffeine, ^c^*p*<0.05 compared with 40mg/Kg/day caffeine.

### Effect of caffeine on oxidant and antioxidant status of the uterus of female Wistar rats

The effect of Caffeine on the Oxidant and Antioxidant Status of the Uterus of Female Wistar Rats is shown in Table 4. Caffeine treatment had a dose-dependent effect on uterine protein levels. While protein levels in rats treated with 10 and 20mg/kg/day caffeine significantly decreased (*p<0.05*) compared to controls, the 40mg/kg/day group displayed a significant increase (*p<0.05*). Similarly, uterine malondialdehyde (MDA) levels significantly increased (*p<0.05*) in the 20 and 40 mg/kg/day groups compared to controls but were then significantly reduced (*p<0.05*) in all withdrawal groups. Catalase activity also exhibited a dose-dependent response, with significant increases (*p<0.05*) in the 20 and 40mg/kg/day groups compared to controls and the 10 mg/kg/day group. However, withdrawal reversed this trend, leading to significant reductions (*p<0.05*) in catalase activity across all caffeine-withdrawn groups. Finally, reduced glutathione (GSH) levels in the uterus followed a contrasting pattern. All caffeine-treated groups displayed no significant changes compared to controls, but withdrawal significantly increased (*p<0.05*) GSH levels in the 10, 20, and 40mg/kg/day groups.

**Table 4:** Effect of caffeine on oxidant and antioxidant status of the uterus of female Wistar rats.

Group	Control	10 mg/Kg/day	20 mg/Kg/day	40 mg/Kg/day	10 mg/Kg/day + Recovery	20 mg/Kg/day + Recovery	40 mg/Kg/day + Recovery
**Protein level (g/dl)**	3.68±0.10	2.87±0.02*	2.85±0.05*	3.24±0.08	3.47±0.20	3.48±0.11	3.88±0.36^ab^
**MDA (U/mg)**	1.23±0.34	4.64±0.57	7.68±1.78*	4.81±0.21*	3.57±0.84^b^	2.14±0.29^c^	1.19±0.41^bc^
**NO (U/mg)**	6.83±1.28	5.17±0.21	5.16±0.15	5.05±0.06	6.40±1.07	5.19±0.37	7.89±1.11
**SOD (U/mg)**	0.67±0.10	0.99±0.06	0.76±0.16	0.83±0.06	0.69±0.09	0.49±0.07	0.74±0.09
**Catalase (IU/L)**	14.3±0.21	18.0±2.41	22.5±1.75*	24.5±2.11*^a^	13.0±0.37^bc^	12.8±0.65^bc^	12.5±0.50^bc^
**GSH (uM/mg)**	0.98±0.32	0.69±0.00	0.73±0.02	0.74±0.04	1.40±0.09^abc^	1.52±0.06^abc^	1.31±0.07^a^

Data are presented as mean ± standard error of mean, n = 5, *p<0.05 compared with control, ^a^*p*<0.05 compared with 10 mg/Kg/day caffeine, ^b^*p*<0.05 compared with 20 mg/Kg/day caffeine, ^c^*p*<0.05 compared with 40mg/Kg/day caffeine.

### Effect of caffeine on female reproductive hormone levels in Wistar rats

**Figure 2:**
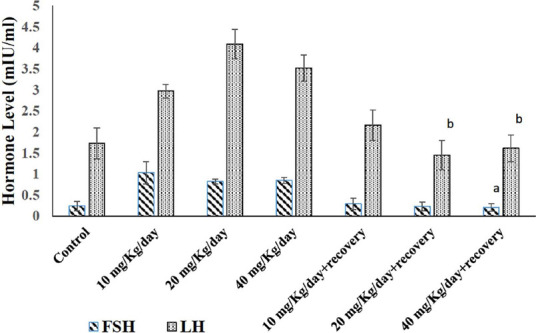
Effects of Caffeine on Estradiol level of female Wistar rats. Columns represent mean±SEM, n = 5, ^a^*p*<0.05 compared to control, ^b^*p*<0.05 compared with 10 mg/Kg/day + recovery.

**Figure 3:**
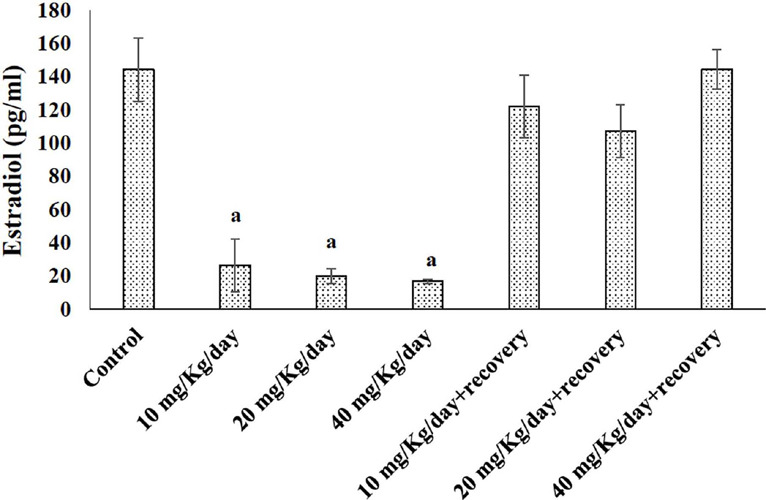
Effects of Caffeine on Estradiol level of female Wistar rats. Columns represent mean±SEM, n = 5, ^a^*p*<0.05 compared to control.

Caffeine withdrawal significantly impacted female hormone levels (*p<0.05*). Compared to rats receiving 10 mg/kg/day caffeine, the 40 mg/kg/day withdrawal group showed a significant decrease (*p<0.05*) in follicle-stimulating hormone (Figure 2). Similarly, luteinizing hormone (Figure 2) levels significantly decreased (*p<0.05*) in the 20 and 40 mg/kg/day withdrawal groups compared to the 10 mg/kg/day caffeine-treated group. Additionally, all caffeine treatment groups (10, 20, and 40 mg/kg/day) displayed significantly lower estradiol levels (*p<0.05*) compared to the control group (Figure 3). Interestingly, these hormonal changes reversed during the withdrawal period.

### Effect of caffeine on the histology of the ovaries, fallopian tubes, and uteri of female Wistar rats

**Figure 4:**
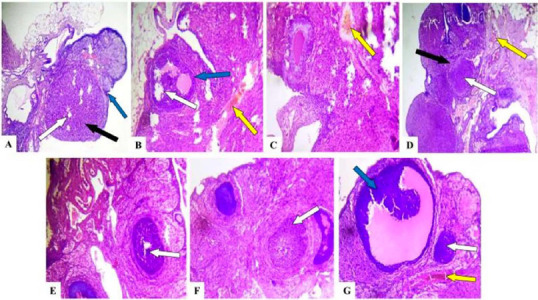
Photomicrograph of ovarian sections of control, caffeine treated, and caffeine withdrawn (recovery) rats. (A) Control (B) 10 mg/Kg/day (C) 20 mg/Kg/day (D) 40mg/Kg/day (E) 10 mg/Kg/day+recovery (F) 20 mg/Kg/day+recovery (G) 40mg/Kg/day+recovery. Note the normal antral follicles (white arrows) with normal theca cells (blue arrows) within the ovarian cortex. Normal ovarian stroma with normal connective tissues (black arrow). The ovarian stroma with congested connective tissues (yellow arrows). Stained by H&E. Magnification: x100.

**Figure 5:**
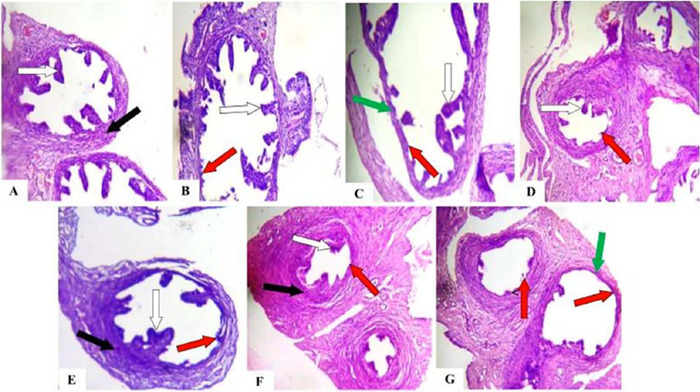
Photomicrograph of fallopian tube sections of control, caffeine treated, and caffeine withdrawn (recovery) rats. (A) Control (B) 10mg/Kg/day (C) 20mg/Kg/day (D) 40mg/Kg/day (E) 10mg/Kg/day+recovery (F) 20mg/Kg/day+recovery (G) 40mg/Kg/day+recovery. Note the ampulla of fallopian tubes with long slender plicae (folds of mucosa) resting on the muscularis (white arrow). Fallopian tubes with the folds of mucosa degenerated off the muscularis (red arrows). Thinning of muscularis (green arrows) Stained by H&E. Magnification: x100.

**Figure 6:**
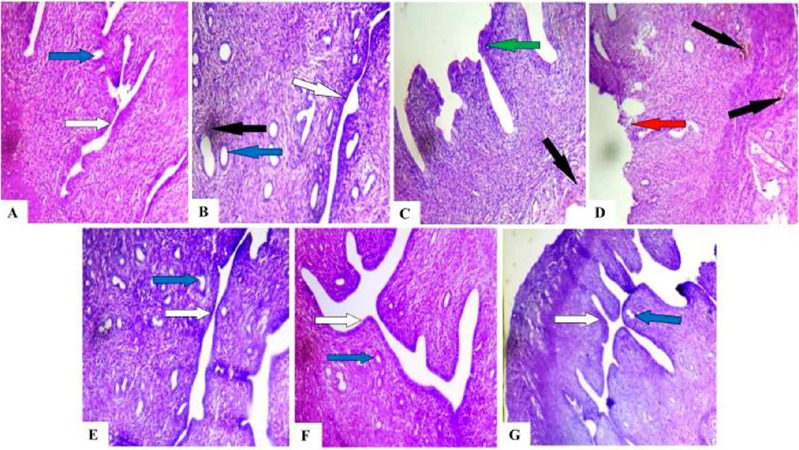
Photomicrograph of uterine sections of control, caffeine treated, and caffeine withdrawn (recovery) rats. (A) Control (B) 10mg/kg/day. (C) 20mg/kg/day. (D) 40mg/Kg/day. (E) 10mg/Kg/day+recovery (F) 20mg/Kg/day+recovery (G) 40mg/Kg/day+recovery. Note the normal endometrium epithelial layer (white arrow), normal endometrial gland (blue arrow), thickened endometrium epithelial layer (green arrow), severe infiltration of inflammatory cells in the stroma of the myometrium (black arrows), degeneration of epithelial layer (red arrow). Stained by H&E. Magnification: x100.

Histological analysis revealed the detrimental effects of caffeine on the reproductive organs. Compared to controls, the ovaries in caffeine-treated groups displayed congested connective tissues in the stroma, with this abnormality persisting even in the 40mg/kg/day withdrawal group (Figure 4). Similarly, the ampulla of the fallopian tubes in all caffeine-treated and withdrawal groups exhibited sloughed plicae resting on the muscularis (Figure 5). The most concerning observation was the severe infiltration of inflammatory cells within the stroma of the uterine myometrium across all caffeine-treated groups (Figure 6). Additionally, the endometrium displayed a thickened epithelial layer in the 20mg/kg/day group and a degenerated epithelial layer in the 40mg/kg/day group compared to controls. These findings suggest that caffeine exposure disrupts the normal architecture of female Wistar rat reproductive organs, with some effects potentially lingering after caffeine withdrawal.

## DISCUSSION

This study investigated the effects of caffeine consumption on the body weight, organ weight, and reproductive function of female Wistar rats. Caffeine treatment resulted in a general decrease in body weight across all groups, with weights increasing again during withdrawal (Westerterp-Plantenga *et al.,* 2005). This aligns with previous findings suggesting caffeine’s ability to promote weight loss through increased sympathetic tone and lipolysis (Harpaz *et al.,* 2017; Van Schaik *et al.,* 2021). This implies that caffeine possibly possesses the ability to reduce body weight and can be used by people who seek to reduce their body weight.

Caffeine treatment also led to a decrease in ovarian and uterine protein levels. This may be due to reduced cell number caused by caffeine-induced cell death or meiosis suppression, as earlier reported by Dorostghoal *et al.* (2011) in a postnatal development study. Li & Winuthayanon (2017) proposed that caffeine may have weakened the muscles in the fallopian tubes, potentially hindering egg transport (Qian *et al.,* 2018). Our findings on reduced protein levels in the fallopian tubes might support this hypothesis. Additionally, Lee *et al.* (2020) observed decreased protein activity in the fallopian tubes of women with high caffeine intake, potentially explaining their longer time to conception (Jurczewska & Szostak-Wçgierek, 2022).

Caffeine, a central nervous system stimulant, readily crosses biological membranes due to its hydrophobic nature (Fredholm *et al.,* 1999). In contrast to previous studies reporting decreased malondialdehyde (MDA) levels with caffeine treatment (Metro *et al.,* 2017; Kaczmarczyk-Sedlak *et al.,* 2019), our study observed increased MDA levels in all organs with increasing caffeine doses. However, MDA levels dropped significantly during withdrawal.

Nitric oxide (NO), synthesized from L-arginine by nitric oxide synthase (NOS), serves as a critical signaling molecule in diverse physiological processes, including immunity, neurotransmission, and vascular function (Mori & Gotoh, 2000). Impaired NO production is associated with various diseases like vascular dysfunction, while its overproduction is linked to conditions like septic shock and neurodegeneration. Interestingly, reduced NO release is considered an early marker of endothelial dysfunction (Mori & Gotoh, 2000). Previous studies reported a decrease in tissue NO after caffeine ingestion, suggesting potential suppressive effects (Bruce *et al.,* 2002; Ferré *et al.,* 2013). However, our findings showed no significant changes in NO levels within the reproductive organs following caffeine treatment.

This discrepancy might be due to several factors: First, unlike previous studies focusing on exhaled NO or skeletal muscle (Corsetti *et al.,* 2007), our investigation examined NO levels within the female reproductive system. NO regulation can vary significantly across different tissues (Schmidt & Walter, 1994). Caffeine might specifically influence NO production pathways in the lungs or skeletal muscles, but not necessarily in the reproductive organs. Secondly, the dose and duration of caffeine exposure can significantly impact NO levels. Previous studies employed acute caffeine administration (Corsetti *et al.,* 2007), whereas our study involved chronic consumption. Chronic exposure might lead to compensatory mechanisms within the reproductive system, maintaining NO homeostasis despite caffeine intake. Furthermore, differences in NO measurement techniques can also contribute to contrasting results. Ours might have focused on total NO levels, while others might have measured specific NO metabolites or isoforms.

The observed decrease in NO levels within the ovary and fallopian tube during the withdrawal phase is intriguing and requires further investigation. It’s possible that chronic caffeine exposure initially upregulates NO production, followed by a compensatory downregulation upon withdrawal. Alternatively, caffeine withdrawal might disrupt the delicate balance of factors influencing NO synthesis within these tissues. The increase in NO levels within the uterus during withdrawal is also noteworthy. This could be a compensatory response to the decreased NO observed in the ovary and fallopian tube, or it might reflect tissue-specific regulatory mechanisms within the uterus itself. Future studies using different NO measurement techniques, a wider range of caffeine doses, and exploring the expression and activity of specific NOS isoforms could shed light on the complex interplay between caffeine and NO regulation within the female reproductive system.

This study investigated the effects of caffeine on antioxidant enzymes (SOD, GSH, and *catalase*) in female rat reproductive organs. Superoxide dismutase (SOD) is the only enzyme that utilizes superoxide anion free radicals as a substrate; superoxide dismutase plays an important role in the metabolism of reactive oxygen species and can stop the damage caused by superoxide anion free radicals (Miao & St Clair, 2009; Wang *et al.,* 2018). Superoxide dismutase (SOD) levels remained unchanged during both treatment and withdrawal phases, aligning with findings by (Liu *et al.,* 2019) but contradicting (Abreu *et al.,* 2011). Catalase catalyzes the conversion of H_2_O_2_ into O_2_ and H_2_O (Weydert & Cullen, 2010; Das & Roychoudhury, 2014). During oxidative stress, cells start to produce energy through a catabolic process, which produces H_2_O_2_ and catalase that can eliminate H_2_O_2_ in an energy-efficient manner (Mallick & Mohn, 2000; Zandi & Schnug, 2022). Catalase showed a notable decrease only during withdrawal. This suggests that caffeine may have maintained catalase activity during treatment, similar to observations by Nilnumkhum *et al.* (2019) who linked caffeine intake to reduced oxidative stress. Glutathione levels increased in the ovary and fallopian tube with both treatment and withdrawal, but not in the uterus. This aligns with Aoyama *et al.* (2011) but disagrees with Verma *et al.* (2010). Increased glutathione could enhance membrane integrity and potentially protect against oxidative damage (Khan *et al.,* 2020).

Female infertility is known to be associated with hormonal imbalances (Lee *et al.,* 2020). While this study observed no significant changes in follicle-stimulating hormone (FSH) and luteinizing hormone (LH) levels during caffeine treatment, a decrease in both hormones was seen during withdrawal, particularly in groups that received higher caffeine doses. This suggests a potential effect of caffeine on the hypothalamic-pituitary-ovarian (HPO) axis, the complex regulatory network governing female reproduction. Caffeine might influence FSH and LH production or secretion through altered ovarian function or disrupted hormone metabolism (Schliep *et al.,* 2015; Bosch *et al.,* 2021).

Elevated FSH levels in women are often indicative of reduced viable egg production (Lee *et al.,* 2020). Conversely, abnormally high LH levels can suggest absent or malfunctioning ovaries (Liu *et al.,* 2012). In this context, the observed decrease in FSH and LH during withdrawal after high-dose caffeine treatment warrants further investigation. It is possible that chronic caffeine exposure initially disrupts the HPO axis, leading to a compensatory downregulation upon withdrawal, causing temporary hormonal suppression.

Furthermore, this study showed a reduction in estradiol levels with caffeine treatment, which reversed during withdrawal. This aligns with findings by Schliep *et al.* (2012) and Wikoff *et al.* (2017) who reported decreased estradiol levels in women consuming caffeinated beverages. Estradiol, a critical sex hormone stimulates follicle growth within the ovary (Chauvin *et al.,* 2022; Perry *et al.,* 2023). Reduced estradiol levels due to caffeine intake could potentially impair folliculogenesis, a crucial step in egg development and ovulation, ultimately impacting fertility.

These findings highlight the potential for caffeine to disrupt hormonal regulation in the female reproductive system. Future studies exploring the mechanisms by which caffeine affects the HPO axis and sex hormone production are warranted to understand the complete picture.

This study observed a severe infiltration of inflammatory cells within the uterine myometrial stroma following caffeine treatment in line with previous reports (Dunselman *et al.,* 2014; Kechagias *et al.,* 2021; Nap & de Roos, 2022). Additionally, the endometrium displayed a thickened epithelial layer in some cases, along with signs of degeneration. These observations suggest a potential detrimental effect of caffeine on uterine tissue integrity and function. Caffeine exposure also induced congested connective tissues within the ovary. This is a histological abnormality previously linked to ovarian cancer development (Franasiak *et al.,* 2021). Interestingly, Terry *et al.* (2007) and Merritt *et al.* (2013) reported a potential association between ovarian cancer risk and genetic variations influencing caffeine metabolism, particularly within the CYP1A1 and CYP1A2 genes encoding cytochrome P450 enzymes responsible for caffeine breakdown. These present findings align with that of (Lueth *et al.,* 2008) who observed a significantly increased risk of ovarian cancer in women consuming caffeinated beverages. However, further investigation is necessary to establish a definitive causal link between caffeine intake and ovarian cancer development. In contrast to the alterations observed in the uterus and ovary, the fallopian tubes displayed a relatively normal appearance. The tubal epithelium and ampullae appeared healthy, with the characteristic folds of tissue (plicae) resting on the muscular layer. This suggests that caffeine might not exert significant detrimental effects on the fallopian tube structure.

## CONCLUSION

This study highlights the potential negative effects of chronic caffeine consumption on female Wistar rat reproductive functions. Caffeine reduces body weight and organ protein levels, potentially via the alteration of oxidant and antioxidant systems. This caused disrupted histological changes in uterine and ovarian tissues thereby impacting fertility. While hormonal alterations were observed during withdrawal, further research is needed to understand the complete picture. With these findings, precautions regarding excessive caffeine intake should be taken by women aiming for pregnancy.
